# Supplementary material for: Understanding hydrogen-bonding structures of molecular crystals via electron and NMR nanocrystallography
Source: Nat Commun. 2019 Aug 6;10:3537. doi: 10.1038/s41467-019-11469-2 (PMC6684599; doi:10.1038/s41467-019-11469-2)
Supplement: Supplementary file 1 — Supplementary Information [file 41467_2019_11469_MOESM1_ESM.pdf]

Supplementary Information

Understanding hydrogen bonding structure of molecular crystals by electron and NMR nano-crystallography

Guzmán-Afonso et al.

Supplementary Note 1. Discussion of the structure determined by ED/SSNMR/GIPAW and previous observation of intra- and intermolecular distances based on SSNMR.

The intramolecular distance between C3(C13) and C9(C19) was reported to be 5.5 Å from the rotational resonance (R_2) curve obtained from using selectively double-^{13}C labeling [1]. While this value disagrees with the distances obtained by the cimetidine form B structure via ED (11.044 Å for I and 7.179 Å for II), the difference comes from the effect of intermolecular interactions. In the NMR study, the intermolecular contact was minimized by mixing the 8-fold excess of natural abundance molecules with doubly ^{13}C labeled molecules which was neglected in the analysis. This is a reasonable assumption when rather short intramolecular ^{13}C-^{13}C distances, i.e. strong ^{13}C-^{13}C dipolar interactions, dominate the decay curve like in form A and M1. However, this assumption is no longer valid in form B, given the negligible intramolecular ^{13}C-^{13}C dipolar interactions; the effect of strong intermolecular interaction should be considered in the analysis. Two short (< 4.0 Å) intermolecular contacts between C3(C13) and C9(C19) are found for each molecule both for I and II. Thus, one fourth of ^{13}C labeled molecule possess the labeled ^{13}C neighboring molecule. This leads to R_2 decay curve with four times smaller than that of the intramolecular ^{13}C-^{13}C proximity (< 4.0 Å). This qualitatively agrees with the four times smaller decay in form B (Fig. 6. in ref [1]) than that in form A and M1 (Fig. 5. in ref [1]).

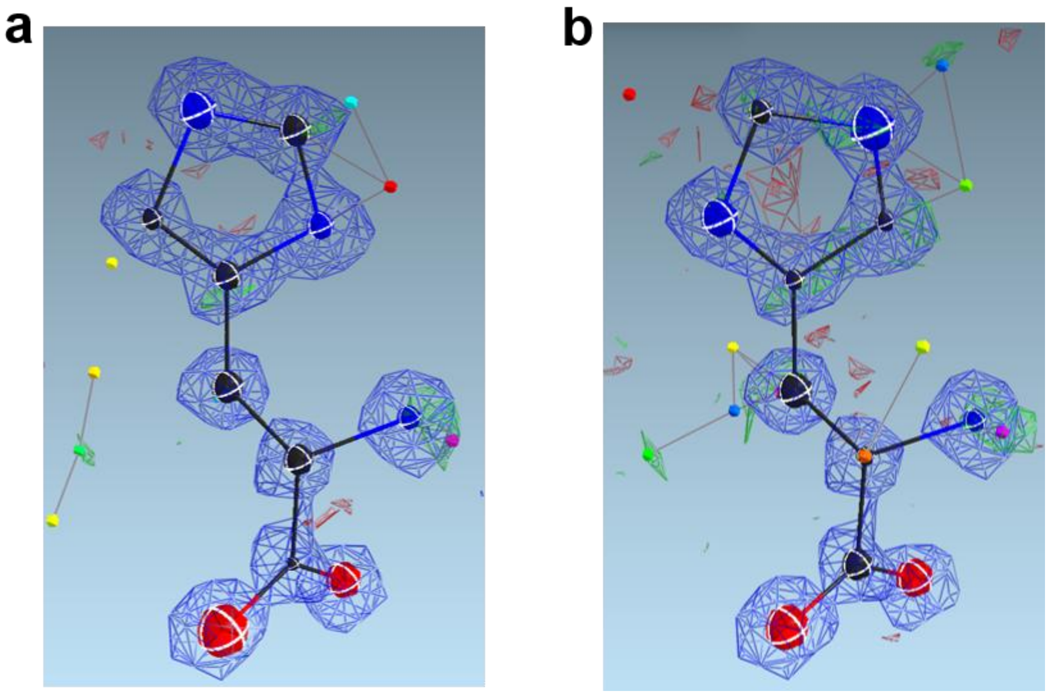

Supplementary Figure 1. Difference potential map (F_o-F_c) and potential map (F_o) of L-histidine. Right after solving the structure using ED, two possible models of L-histidine (LH) can be considered as shown in **a** LH1(2) and **b** LH3(4) based on only non-hydrogen atoms. The LH1(2) and LH3(4) is the same as the L-histidine structure of Fig. 2b(c) and Fig. 2d(e) in main text. The LH1-4 indicate the L-histidine structure of Fig. 2b-d in main text. SHELXL displays the F_o map at 1.2 e Å^{-3} (blue mesh) and the F_o-F_c map at 0.54 e Å^{-3} (green and red mesh). Small icosahedra represent maxima in the F_o-F_c map and the peak heights are represented by rainbow color from purple (high) to red (low). The icosahedra with a superimposed green F_o-F_c map (positive density) indicate the possible positions of the hydrogen atoms. ADPs (atomic displacement parameters) for non-hydrogen atoms are at 50% probability level. The red, blue, black, and white atoms denote oxygen, nitrogen, carbon, and hydrogen atoms, respectively.

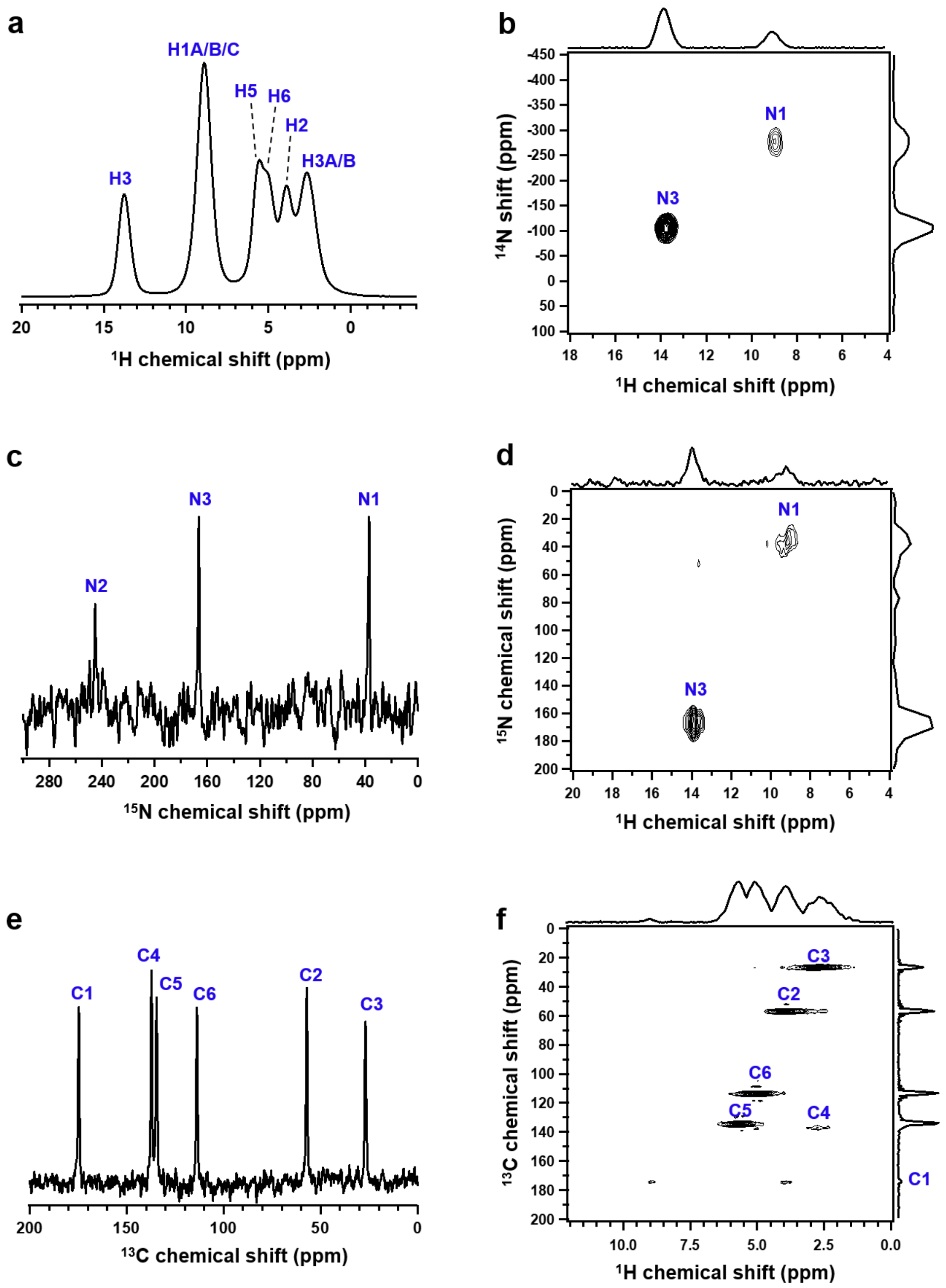

Supplementary Figure 2. ^{1}H, ^{14}N, ^{15}N, and ^{13}C SSNMR spectra of L-histidine. **a** 1D ^{1}H single-pulse spectrum and **b** 2D ^{1}H-^{14}N heternuclear multi-quantum correlation SSNMR spectrum. **c** 1D ^{15}N CPMAS and **d** 2D ^{1}H-^{15}N HETCOR SSNMR spectrum. **e** 1D ^{13}C CPMAS and **f** 2D ^{1}H-^{13}C HETCOR SSNMR spectrum. The peak assignments are based on the LH1 structure in Fig. 2b. The chemical assignment is identical to the previous reported results [2].

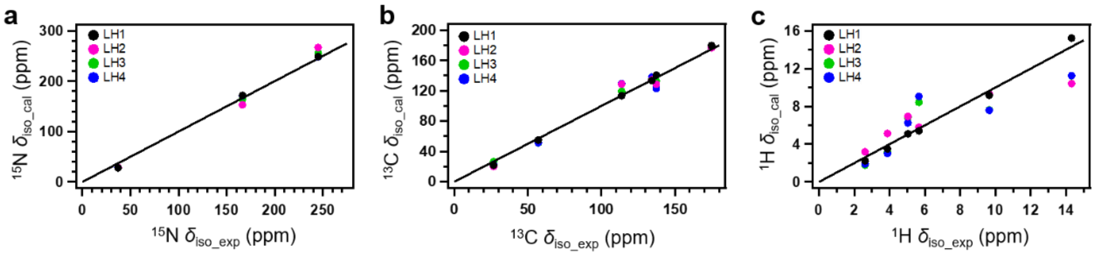

Supplementary Figure 3. Comparison of the experimental (δ_{iso_exp}) and the GIPAW-calculated isotropic chemical shift (δ_{iso_cal}) of L-histidine. **a** ^{15}N, **b** ^{13}C, and **c** ^{1}H chemical shifts.

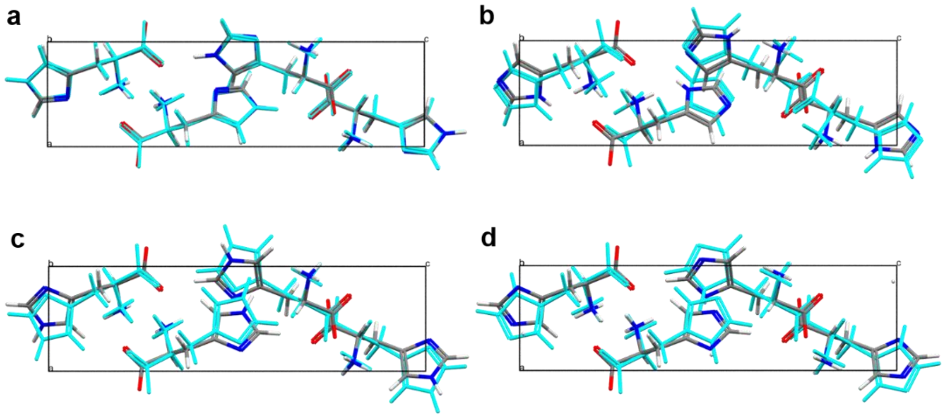

Supplementary Figure 4. Comparison of L-histidine structures solved by ED/SSNMR and optimized by GIPAW calculation (sky-blue molecules). The structures of **a** LH1, **b** LH2, **c** LH3, and **d** LH4, are displayed in the *ac* plane.

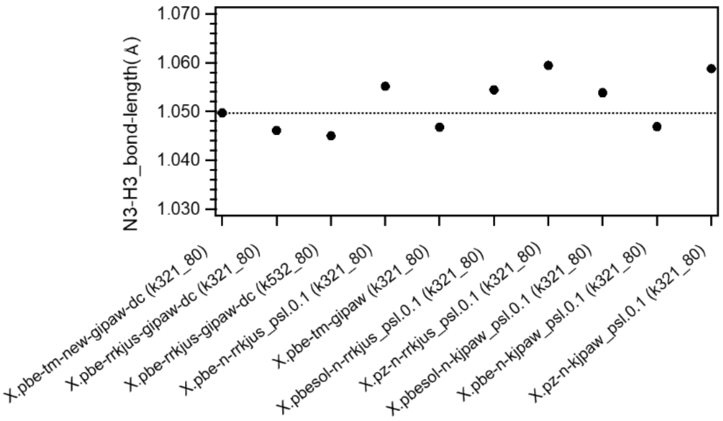

Supplementary Figure 5. N3-H3 bond lengths of LH1 as a function of pseudopotentials. The broken line (1.05 Å of N3-H3) represents our choice of pseudopotentials (X.phe-tm-new-gipaw-dc (k321_80).

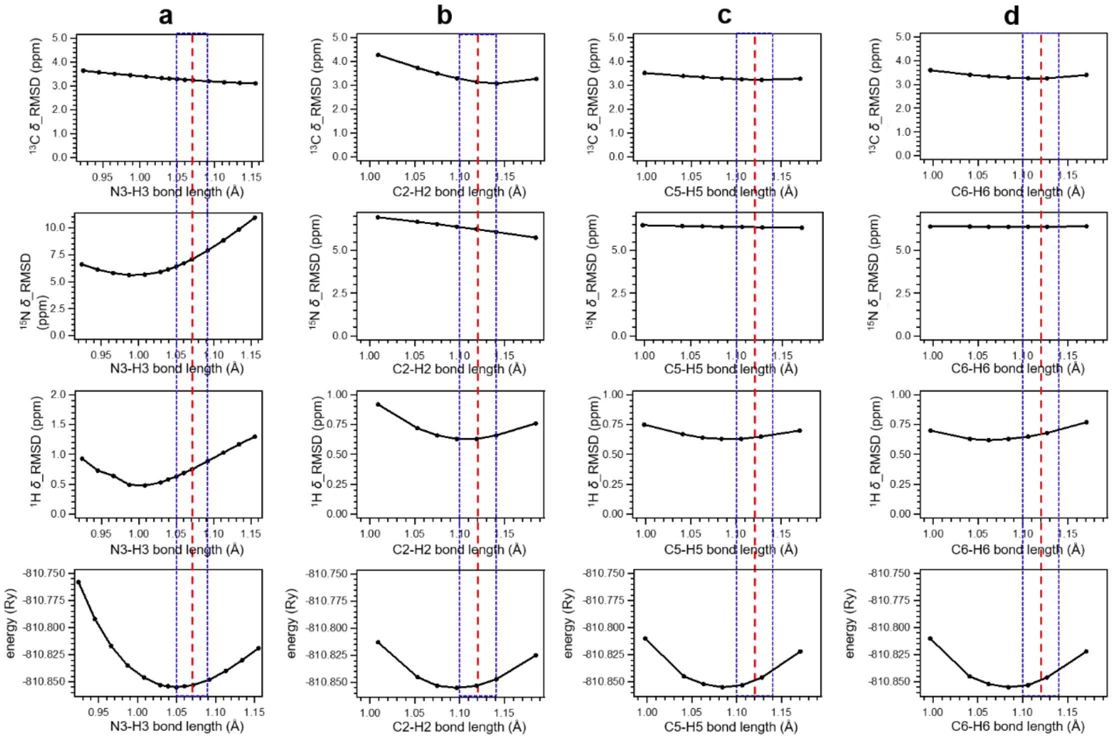

Supplementary Figure 6. Chemical shift RMSD values and energy as a function of X-H bond lengths. Chemical shift (δ) RMSD values of ^{1}H, ^{13}C, and ^{15}N and energy are calculated as a function of **a** N3-H3, **b** C2-H2, **c** C5-H5, and **d** C6-H6 bond lengths. The RMSD values were obtained between the SSNMR experimental and GIPAW-calculated isotropic chemical shifts. Broken red vertical lines represent the bond length measured by SSNMR and broken blue lines are standard uncertainties. The standard uncertainty is calculated by the full width of half maximum of SSNMR peak.

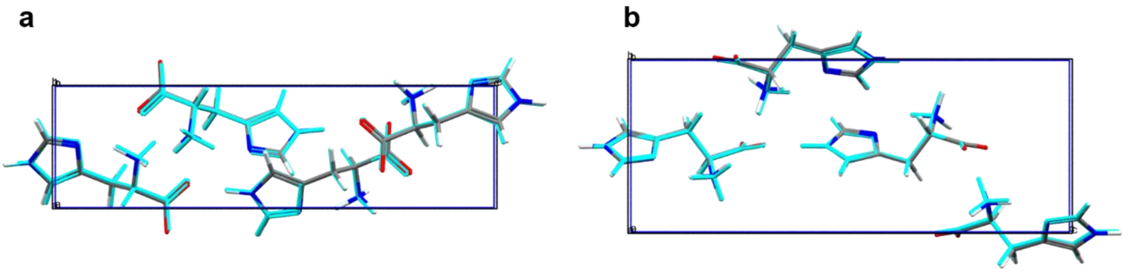

Supplementary Figure 7. Comparison of L-histidine solved by ED/SSNMR/GIPAW and by single crystal neutron diffraction (sky-blue)[3]. The structures are displayed in **a** *ac* and **b** *bc* (right) plane.

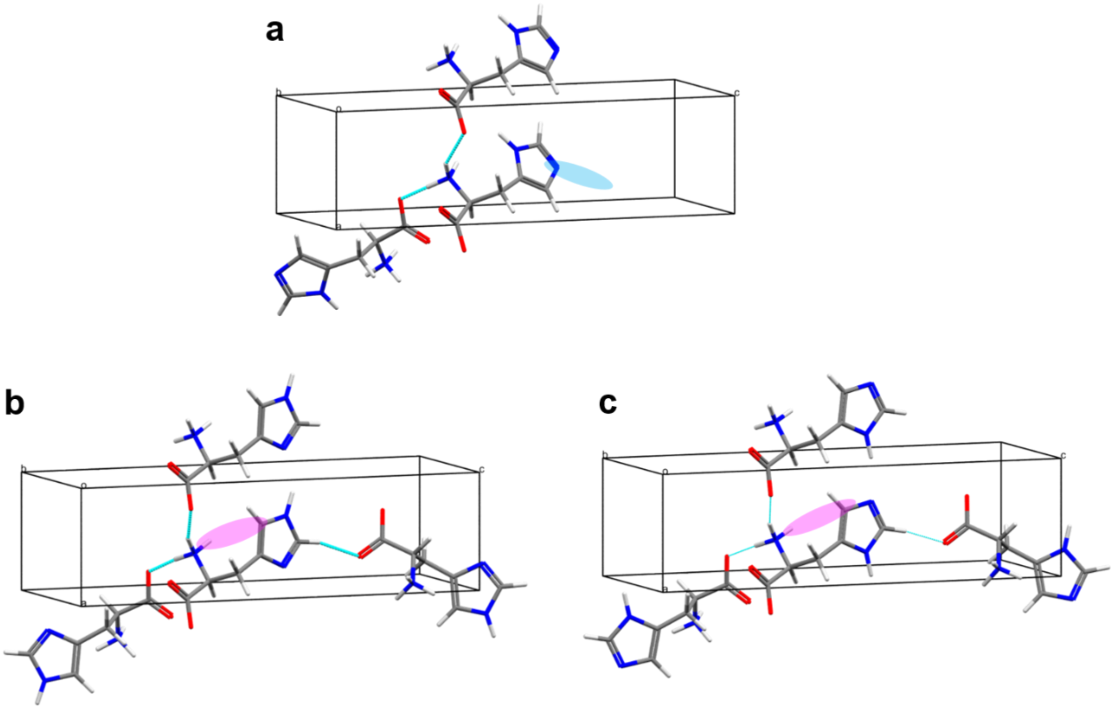

Supplementary Figure 8. Hydrogen-bonding network of L-histidine (LH). The unit cell consists of the adjacent molecules of **a** LH2, **b** LH3, and **c** LH4 through the inter-hydrogen bonds. The hydrogen bonds are displayed by the sky-blue dotted lines. The sky-blue and magenta circles denote the missing inter- and intra-hydrogen bonds, respectively.

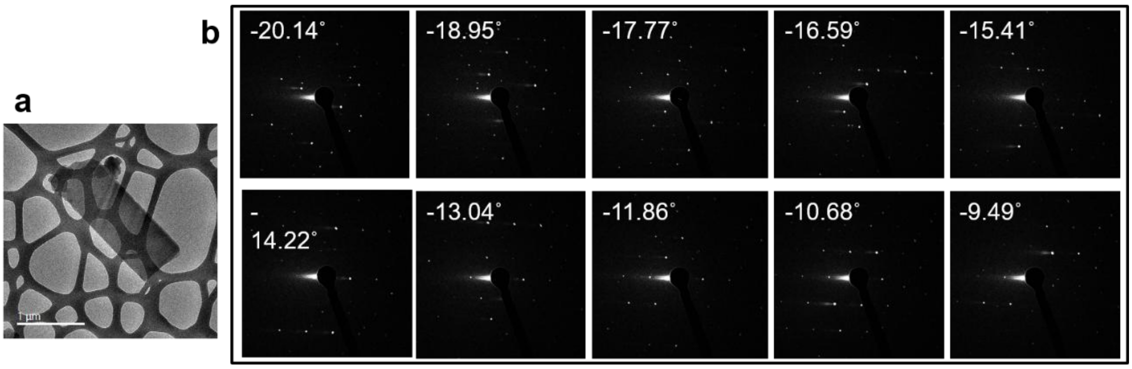

Supplementary Figure 9. TEM image and a series of ED patterns of microcrystal cimetidine form B. **a** TEM image of a cimetidine form B crystal on a thin carbon film. It was obtained right after ED measurement. **b** Selected ED patterns of cimetidine form B crystal under continuous rotation. The number of each frame represents a starting rotation angle of each ED patterns. Each diffraction pattern was obtained every 1.18˚.

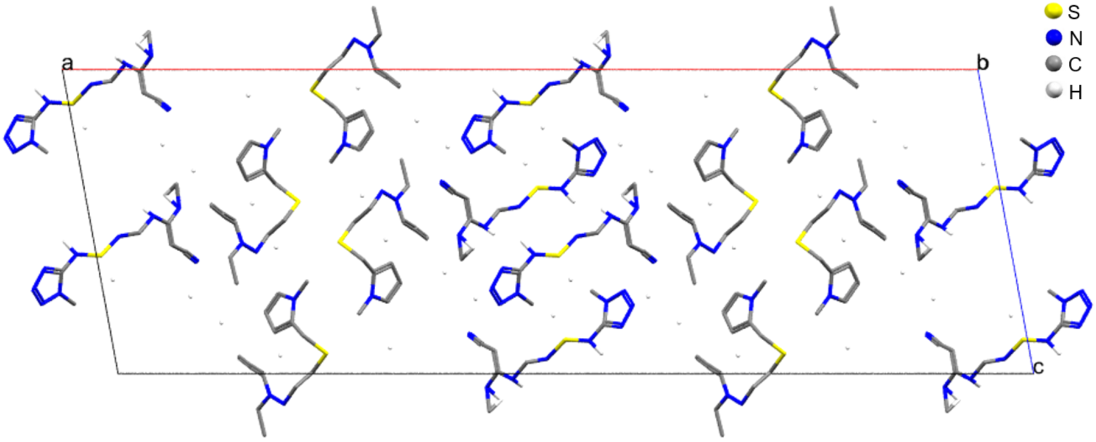

Supplementary Figure 10. Cimetidine form B crystal structure right after phasing by the SIR2014 software. It is displayed in the *ac* plane and the R factor is 20.02 %. The yellow, blue, grey, and white atoms denote sulfur, nitrogen, carbon, and hydrogen atoms, respectively.

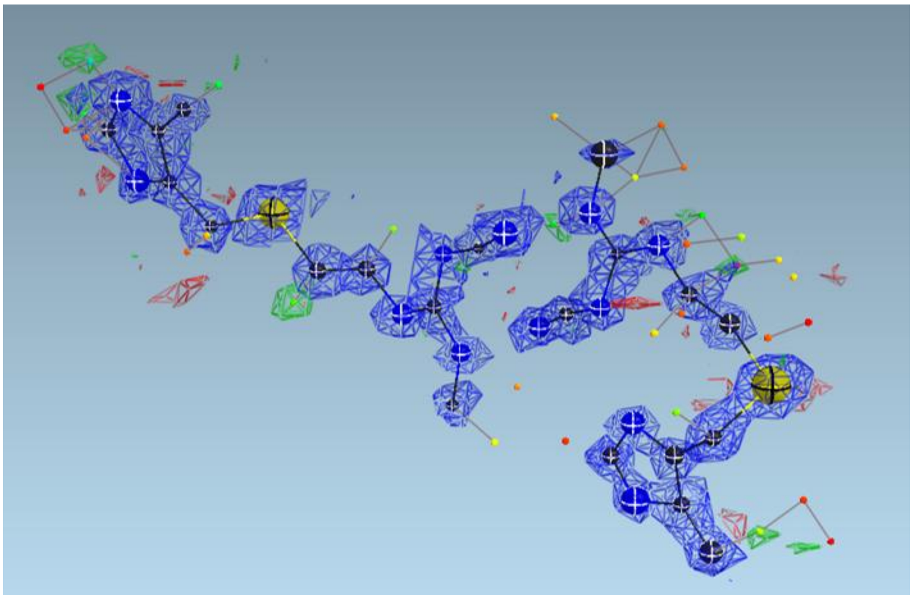

Supplementary Figure 11. Difference potential map (F_o-F_c) and potential map (F_o) of cimetidine form B. SHELXL displays the F_o map at 0.54 e Å^{-3} (blue mesh) and the F_o-F_c map at 0.29 e Å^{-3} (green and red mesh). Small icosahedra represent maxima in the F_o-F_c map and the peak heights are represented by rainbow color from purple (high) to red (low). The icosahedra with a superimposed green F_o-F_c map (positive density) indicate the possible positions of the hydrogen atoms. ADPs for non-hydrogen atoms are at 50% probability level. The yellow, blue, black, and white atoms denote the sulfur, nitrogen, carbon, and hydrogen atoms.

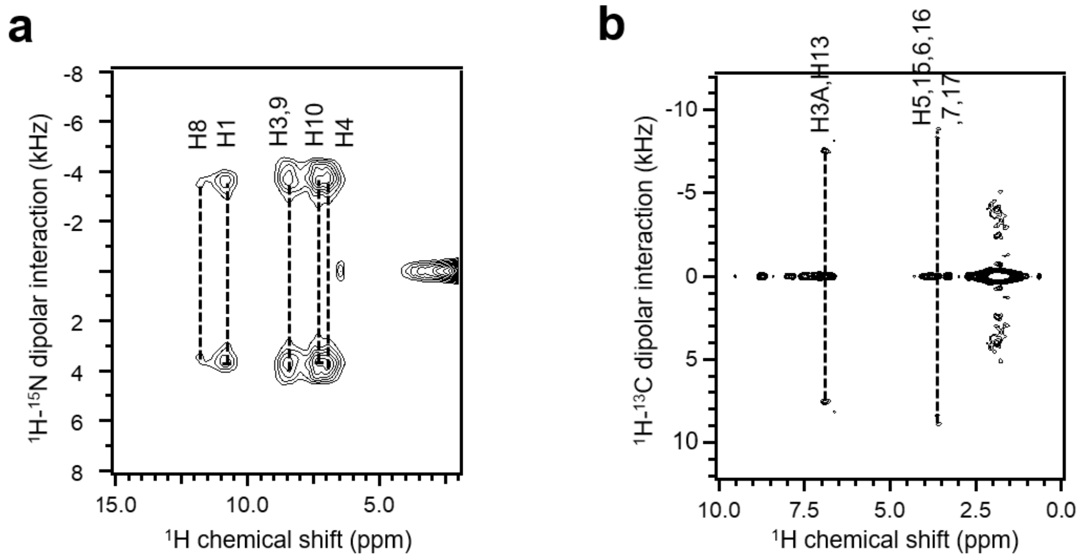

Supplementary Figure 12. Cimetidine form B bond lengths measured by SSNMR. **a** 2D ^{1}H-^{15}N invCP-VC spectrum. The dipolar coupling is 7.05 kHz at ^{1}H chemical shift (δ_{1H}) of 11.73 ppm (H8), 7.27 kHz at δ_{1H} = 10.87 ppm (H1), 7.61 kHz at δ_{1H} = 8.60 ppm (H3, 9), 7.72 kHz at δ_{1H} = 7.38 ppm (H10), and 7.50 kHz at δ_{1H} = 6.96 ppm (H4). **b** 2D ^{1}H-^{13}C invCP-VC spectrum. The dipolar coupling is 15.14 kHz at δ_{1H} = 6.81 ppm (H3A, H13) and 17.77 kHz at δ_{1H} = 3.4 ppm (H5, 15, 6, 16, 7, 17). The total number of scans was 48 for ^{1}H-^{13}C and 116 for ^{1}H-^{15}N invCP-VC spectra.

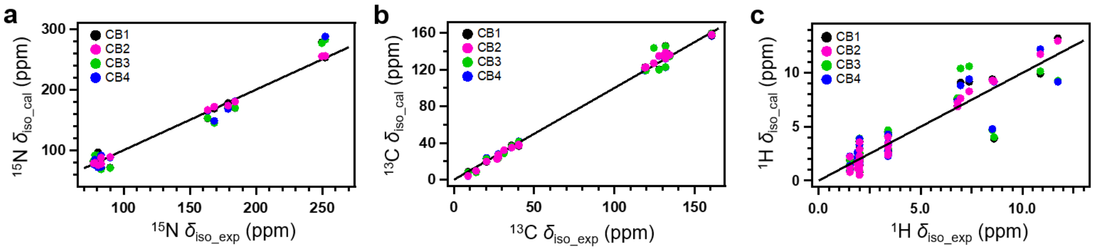

Supplementary Figure 13. Comparison of the experimental (δ_{iso_exp}) and the GIPAW-calculated isotropic chemical shift (δ_{iso_cal}) of cimetidine form B. **a** ^{15}N, **b** ^{13}C, and **c** ^{1}H chemical shifts.

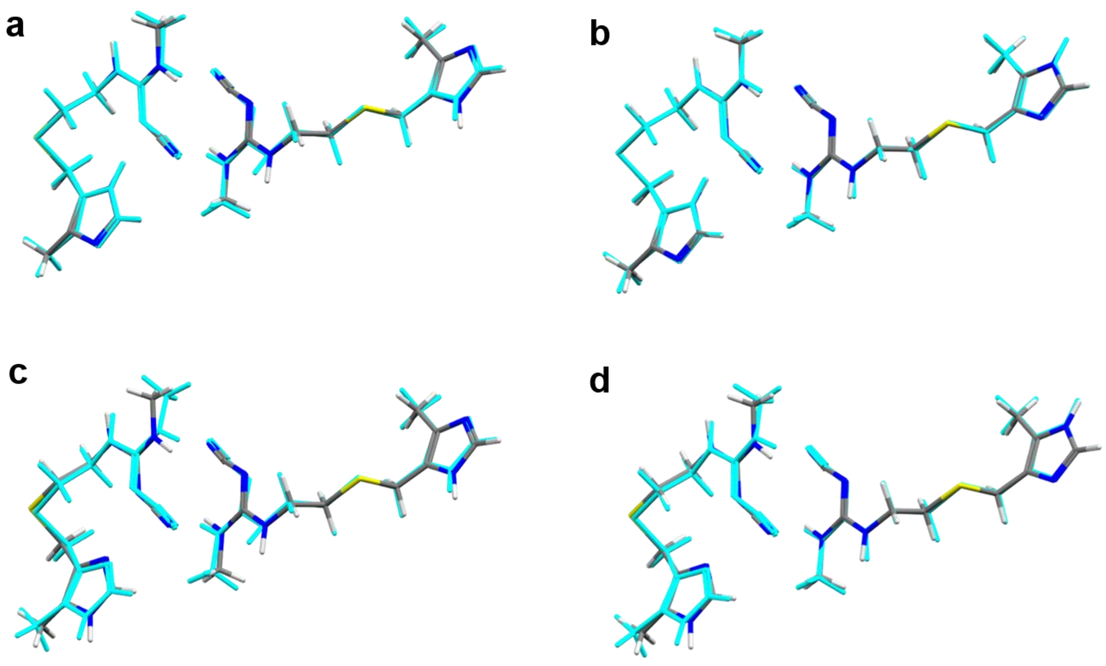

Supplementary Figure 14. Comparison of cimetidine form B structures solved by ED/SSNMR and optimized by GIPAW calculation (sky-blue molecules). The structures of **a** CB1, **b** CB2, **c** CB3, and **d** CB4 are displayed in the *ac* plane.

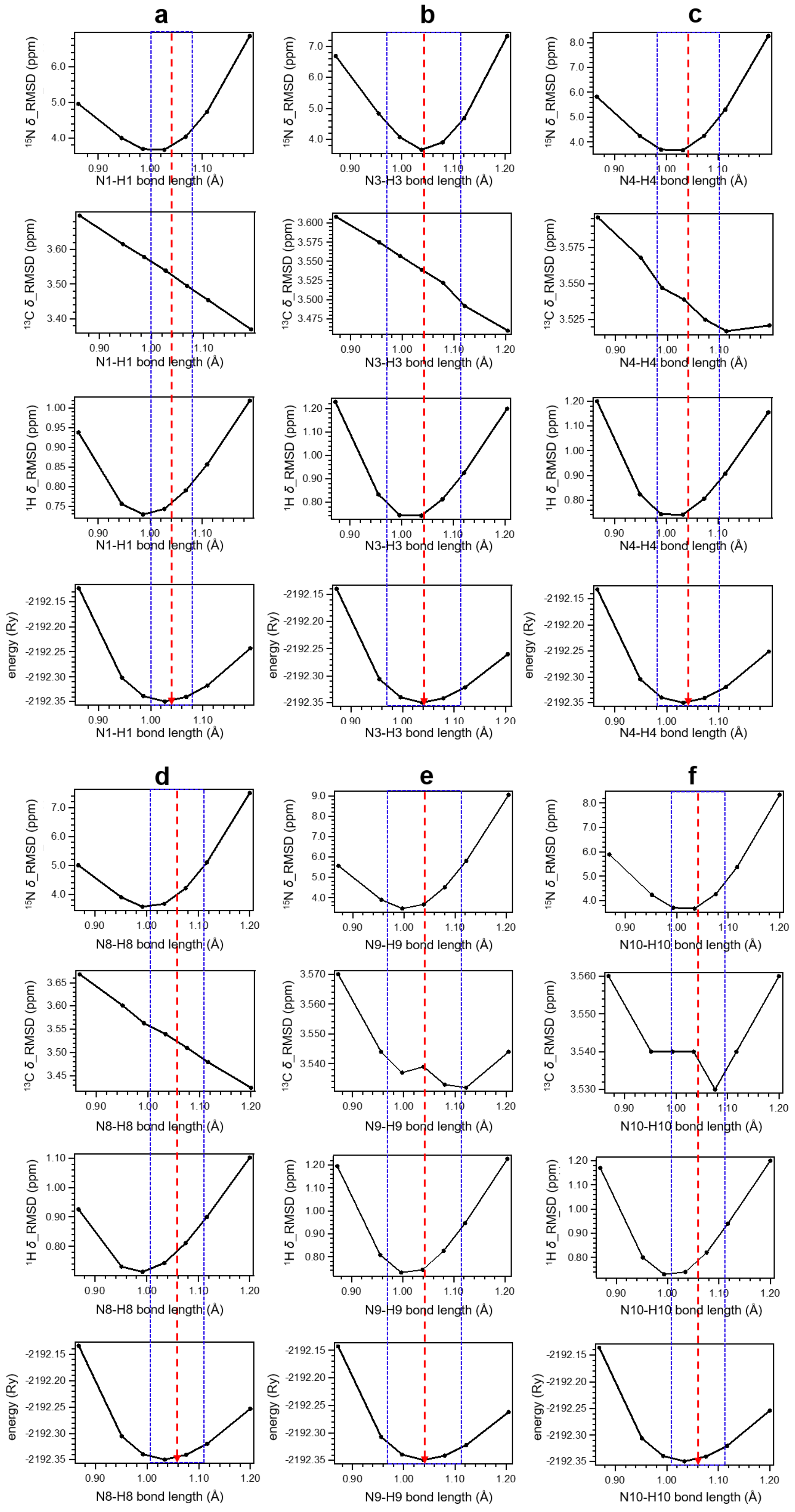
a
b
c
d
e
f
15N δ_RMSD (ppm)
13C δ_RMSD (ppm)
1H δ_RMSD (ppm)
energy (Ry)
N1-H1 bond length (Å)
N3-H3 bond length (Å)
N4-H4 bond length (Å)
N8-H8 bond length (Å)
N9-H9 bond length (Å)
N10-H10 bond length (Å)

Supplementary Figure 15. Chemical shift RMSD values and energy as a function of X-H bond lengths. The chemical shift (δ) RMSD values and energy are calculated as a function of **a** N1-H1, **b** N3-H3, **c** N4-H4, **d** N8-H8, **e** N9-H9, **f** N10-H10 bond lengths from left to right column. Broken red vertical lines represent the bond length measured by SSNMR and broken blue lines are standard uncertainties. The standard uncertainty is calculated by the full width of half maximum of SSNMR peak.

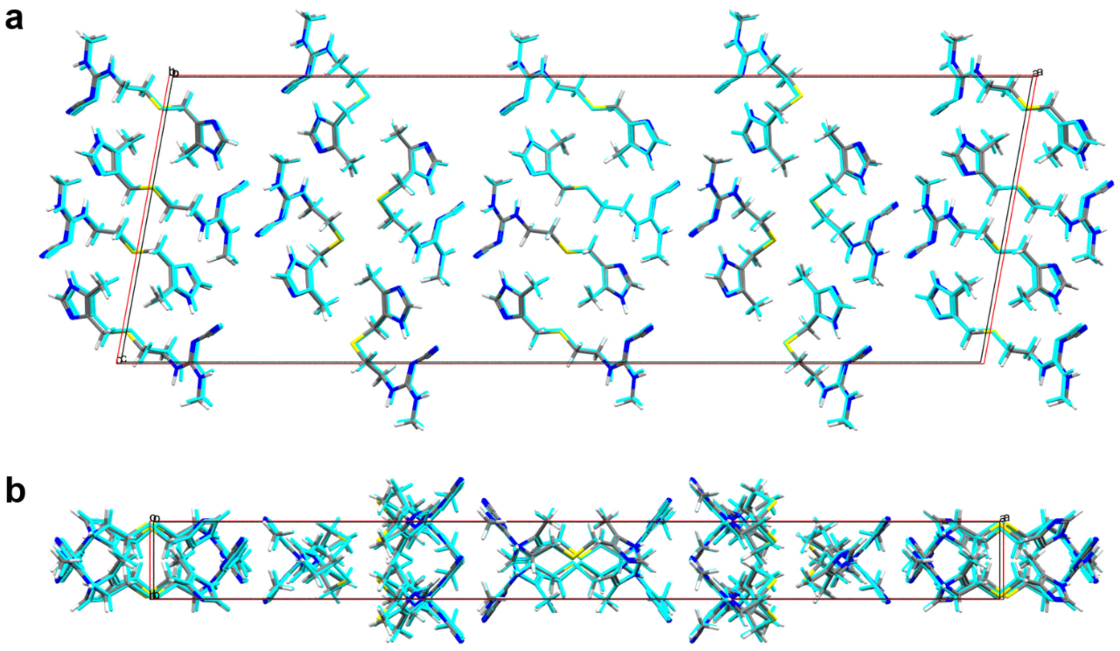

Supplementary Figure 16. Comparison of cimetidine form B solved by ED/SSNMR/GIPAW and by SCXRD (sky-blue). The structures are displayed in **a** *ac* and **b** *ab* plane.

Supplementary Table 1. Characteristics of individual data sets for L-histidine The data were collected by XDS software, all data sets were merged by BLEND program of CCP4 software, and solved by the SIR2014 software for the crystal structure. The individual set of data was obtained from the different crystals.

**Data set**	1	2	3	4	5
No. of frames	33	19	30	32	82
Range of data collection (˚)	-34.47 ~ -14.38	-30.96 ~ 10.39	-25.71 ~ -4.36	-14.10 ~ 20.16	-29.96 ~ 6.66
Rotation angle (˚)	1.1815	1.1815	1.1857	1.1814	1.1814

**Data collection by XDS**					
Space group	$P2_12_12_1$	$P2_12_12_1$	$P2_12_12_1$	$P2_12_12_1$	$P2_12_12_1$
a (Å)	5.20	5.30	5.20	5.30	5.30
b (Å)	7.50	7.50	7.40	7.50	7.40
c (Å)	19.10	19.00	18.90	18.90	19.00
Resolution range (Å)	4.85-0.99	4.22-0.88	5.01-0.94	9.45-0.91	7.40-0.97
No. of reflections observed	559	343	545	636	865
No. of reflections unique	263	162	261	302	349
No. of reflections possible	515	703	568	646	544
Completeness of data (%)	51.1	23.0	46.0	46.7	64.2
R factor observed (%)	30.7	14.3	20.4	8.4	22.7
R factor expected (%)	31.4	14.6	21.1	9.5	23.8
I/σ(I)	2.20	4.25	3.81	6.74	2.73
R meas (%)	38.3	18.7	26.4	10.7	28.7
CC(1/2)	81.0	98.1	94.9	99.0	94.0

**Blending data by ccp4i and Solving structure by SIR2014**	
No. of frames	196
Resolution range (Å)	9.50-1.01
No. of reflections observed	417
Completeness of data (%)	91.20
R meas (%)	31.6
R factor (%)	20.86

Supplementary Table 2. **Crystallographic data of L-histidine crystal determined by ED and ND** The ND data were obtained from the previously reported results [3].

L-histidine	ED	ND
Chemical formula	C6 H9 N3 O2	C6 H9 N3 O2
Molecular weight	155.16	155.16
Measurement temperature (K)[a]	96(2)	295
Space group	$P2_12_12_1$	$P2_12_12_1$
Crystal symmetry	orthorhombic	orthorhombic
a, b, c (Å)	5.27(5), 7.44(5), 18.99(8)	5.175, 7.345, 18.750
α, β, γ (˚)	90, 90, 90	90, 90, 90
Z	4	4
V (Å^3)	745(9)	709.784
Reflection total number	417	***
R factor (%)	19.81	5.8

Supplementary Table 3. *R* factors (%) for the each refinement step The SHELXL software refined L-histidine structures.

Refinement factors	LH1	LH2	LH3	LH4
Initial value	20.86			
Elimination of H atoms	26.27			
Refinement of atomic positions	22.71	22.71	23.86	23.86
Addition of H atoms	20.09	20.41	21.28	21.21
Restraint of bond length	20.06	20.34	21.43	21.21
WGHT	19.81	20.00	21.06	21.76

Supplementary Table 4. Bond lengths for all structures of L-histidine The lengths were obtained by standard geometrical constraint with the SHELXL software, by SSNMR experiment, and by GIPAW calculation. The bond lengths in the column of SHELXL, SSNMR, and GIPAW were determined at T = 96.15, 303.15, and 0 K, respectively. The standard uncertainties in parentheses in the column of SSNMR are calculated from the width at half maximum of the peaks of the slice data extracted along dipolar coupling dimension.

			LH1	LH2	LH3	LH4
Å	SHELXL	SSNMR	GIPAW	GIPAW	GIPAW	GIPAW
N2-H2A		1.07 (±0.02)		1.017		1.037
N3-H3	0.880	1.07 (±0.02)	1.050		1.033	
C2-H2	1.000	1.12 (±0.02)	1.097	1.096	1.096	1.094
C5-H5	0.950	1.12 (±0.02)	1.084	1.083	1.076	1.081
C6-H6	0.950	1.12 (±0.02)	1.084	1.083	1.089	1.090

Supplementary Table 5. ^{1}H, ^{13}C, and ^{15}N isotropic chemical shifts for all L-histidine structures The values were obtained from SSNMR experiments ($\delta_{\mathrm{iso_exp}}$) and GIPAW calculation ($\delta_{\mathrm{iso_cal}}$).

^{1}H	$\delta_{\mathrm{iso\_exp}}$ (ppm)	LH1	LH2	LH3	LH4
		$\delta_{\mathrm{iso\_calc}}$ (ppm)	$\delta_{\mathrm{iso\_calc}}$ (ppm)	$\delta_{\mathrm{iso\_calc}}$ (ppm)	$\delta_{\mathrm{iso\_calc}}$ (ppm)
H1A	9.64	9.44	15.17	8.15	7.96
H1B	9.64	10.33	5.50	13.26	13.30
H1C	9.64	8.35	7.46	3.74	3.77
H2	3.87	3.69	5.17	3.92	3.79
H2A	14.31		10.45		12.01
H3	14.31	15.42		12.00	
H3A	2.6	2.48	2.62	2.48	2.53
H3B	2.6	2.39	3.83	2.61	2.78
H5	5.66	5.61	5.83	9.19	9.81
H6	5.03	5.29	6.97	7.60	7.01

^{15}N	$\delta_{\mathrm{iso\_exp}}$ (ppm)	LH1	LH2	LH3	LH4
		$\delta_{\mathrm{iso\_calc}}$ (ppm)	$\delta_{\mathrm{iso\_calc}}$ (ppm)	$\delta_{\mathrm{iso\_calc}}$ (ppm)	$\delta_{\mathrm{iso\_calc}}$ (ppm)
N1	37.00	28.03	28.79	28.05	29.02
N2	166.42		152.99		171.30
N2	245.15	248.67		256.06	
N3	166.42	171.86		164.46	
N3	245.15		266.78		248.26

^{13}C	$\delta_{\mathrm{iso\_exp}}$ (ppm)	LH1	LH2	LH3	LH4
		$\delta_{\mathrm{iso\_calc}}$ (ppm)	$\delta_{\mathrm{iso\_calc}}$ (ppm)	$\delta_{\mathrm{iso\_calc}}$ (ppm)	$\delta_{\mathrm{iso\_calc}}$ (ppm)
C1	174.81	179.65	176.65	178.77	178.68
C2	57.09	55.06	55.10	51.39	51.54
C3	26.73	21.69	20.17	26.26	23.07
C4	137.31	140.65	128.69	132.93	122.97
C5	134.32	133.49	134.53	135.45	138.25
C6	113.73	113.44	128.88	119.19	129.51

Supplementary Table 6. Characteristics of individual data sets for cimetidine form B The data were collected by XDS software, all data sets were merged by BLEND program of CCP4 software, and solved by the SIR2014 software for the crystal structure. The individual set of data was obtained from the different crystals.

**Data set**	1	2	3
No. of frames	45	40	26
Range of data collection (˚)	-31.96 ~ 11.79	-35.97 ~ 10.33	-29.32 ~ 5.02
Precession angle (˚)	1.1825	1.1872	1.1838

**Data collection by XDS**			
Space group	*C*2/*c*	*C*2/*c*	*C*2/*c*
a (Å)	55.30	55.60	55.50
b (Å)	5.00	5.00	5.55
c (Å)	18.70	18.70	18.80
β (˚)	100.90	100.00	100.2
Resolution range (Å)	10.05-0.84	9.23-0.85	7.10-0.88
No. of reflections observed	3161	2827	1758
No. of reflections unique	1380	1466	1029
No. of reflections possible	4459	4327	3930
Completeness of data (%)	30.9	33.9	26.2
R factor observed (%)	18.9	16.0	14.5
R factor expected (%)	21.4	16.2	16.1
I/σ(I)	2.58	2.79	2.68
R meas (%)	23.8	21.4	20.4
CC(1/2)	97.3	95.5	96.4

**Blending data by ccp4i and Solving structure by SIR2014**	
No. of frames	111
Resolution range (Å)	8.36-1.06
No. of reflections observed	1523
Completeness of data (%)	69.90
R meas (%)	25.3
R factor (%)	20.02

Supplementary Table 7. Crystallographic data of cimetidine form B crystal determined by ED and SCXRD

Cimetidine form B	ED	SCXRD
Chemical formula	C10 H16 N6 S	C10 H16 N6 S
Molecular weight	252.35	252.35
ED measurement temperature (K)	96(2)	90
Crystal symmetry	monoclinic	monoclinic
Space group	*C*2/*c*	*C*2/*c*
a, b, c (Å)	55.45(15), 5.000, 18.72(6)	54.938, 4.896, 18.523
α, β, γ (˚)	90, 100.4(5), 90	90, 100.297, 90
Z	16	16
V (Å^3)	5105(23)	4901.8
Reflection total number	1523	4320
R factor (%)	19.69	11.85

Supplementary Table 8. *R* factors (%) for the each refinement step The SHELXL software refined cimetidine form B structures.

Refinement factors	CB1	CB2	CB3	CB4
Initial value	20.02			
Elimination of H atoms	23.74			
Refinement of atomic positions	22.56			
Addition of H atoms	20.06	19.79	20.26	19.96
Restraint of bond length	19.96	19.64	20.19	19.89
WGHT	19.96	19.69	20.13	19.87

Supplementary Table 9. Bond lengths for all structures of cimetidine form B The lengths were obtained by standard geometrical constraint with the SHELXL software, by SSNMR experiment, and by GIPAW calculation. The bond lengths in the column of SHELXL, SSNMR, and GIPAW were determined at T = 96.15, 303.15, and 0 K, respectively. The standard uncertainties in parentheses in the column of SSNMR are calculated from the width at half maximum of the peaks of the slice data extracted along dipolar coupling dimension.

			CB1	CB2	CB3	CB4
	SHELXL	SSNMR	GIPAW	GIPAW	GIPAW	GIPAW
N1-H1	0.880	1.06 (±0.04)		1.026		1.027
N2-H2	0.880	1.06 (±0.04)	1.019		1.019	
N3-H3	0.880	1.04 (±0.07)	1.018	1.038	1.019	1.037
N4-H4	0.880	1.04 (±0.06)	1.036	1.031	1.042	1.036
N7-H7	0.880	1.06 (±0.05)			1.017	1.017
N8-H8	0.880	1.06 (±0.05)	1.034	1.033		
N9-H9	0.880	1.04 (±0.07)	1.039	1.039	1.015	1.014
N10-H10	0.880	1.04 (±0.05)	1.038	1.034	1.046	1.039
C3-H3A	0.950	1.12 (±0.01)	1.085	1.083	1.085	1.083
C5-H5A	0.990	1.06 (±0.01)	1.100	1.096	1.100	1.096
C5-H5B	0.990	1.06 (±0.01)	1.099	1.099	1.0993	1.0994
C6-H6A	0.990	1.06 (±0.01)	1.0996	1.0995	1.0996	1.0998
C6-H6B	0.990	1.06 (±0.01)	1.101	1.1	1.101	1.099
C7-H7A	0.990	1.06 (±0.01)	1.0965	1.0974	1.0964	1.0971
C7-H7B	0.990	1.06 (±0.01)	1.099	1.095	1.099	1.095
C13-H13	0.950	1.12 (±0.01)	1.084	1.084	1.085	1.084
C15-H15A	0.990	1.06 (±0.01)	1.097	1.097	1.097	1.097
C15-H15B	0.990	1.06 (±0.01)	1.098	1.098	1.095	1.095
C16-H16A	0.990	1.06 (±0.01)	1.097	1.098	1.099	1.099
C16-H16B	0.990	1.06 (±0.01)	1.096	1.096	1.095	1.095
C17-H17A	0.990	1.06 (±0.01)	1.097	1.097	1.096	1.097
C17-H17B	0.990	1.06 (±0.01)	1.097	1.097	1.099	1.099

Supplementary Table 10. ^{1}H, ^{13}C, and ^{15}N isotropic chemical shifts for all cimetidine form B structures The values were obtained from SSNMR experiments (δ_{iso_exp}) and GIPAW calculation (δ_{iso_cal}).

^{1}H	δ_{iso_exp} (ppm)	CB1	CB2	CB3	CB4
		δ_{iso_calc} (ppm)	δ_{iso_calc} (ppm)	δ_{iso_calc} (ppm)	δ_{iso_calc} (ppm)
H1A	1.53	1.85	2.20	1.94	2.26
H1B	1.53	0.88	0.79	0.99	0.86
H1C	1.53	1.41	1.22	1.47	1.39
H1	10.87		11.72		12.19
H2	10.87	9.91		10.15	
H3A	6.81	7.54	7.22	7.67	7.38
H3	8.60	3.88	9.20	4.04	9.17
H4	6.96	9.11	7.63	10.41	8.83
H5A	3.40	4.07	3.40	4.06	3.48
H5B	3.40	4.52	4.05	4.69	4.24
H6A	3.40	2.83	2.37	2.91	2.43
H6B	3.40	2.89	3.13	2.98	3.30
H7A	3.40	2.31	2.57	2.52	2.74
H7B	3.40	4.53	3.37	4.42	3.38
H7	11.73			9.26	9.16
H8	11.73	13.22	12.96		
H9A	2.00	0.80	0.53	0.84	0.54
H9B	2.00	1.84	1.47	1.81	1.52
H9C	2.00	2.08	1.74	2.52	1.98
H9	8.52	9.42	9.30	4.69	4.79
H10	7.38	9.17	8.28	10.60	9.42
H11A	1.90	1.12	1.12	1.97	1.92
H11B	1.90	1.54	1.51	2.58	2.61
H11C	1.90	1.81	1.72	2.20	2.12
H13	6.81	6.95	6.87	7.38	7.38
H15A	2.00	1.99	1.89	1.72	1.68
H15B	2.00	3.68	3.57	3.92	3.88
H16A	2.00	2.48	2.29	1.94	1.87
H16B	2.00	3.02	2.89	3.60	3.32
H17A	2.00	3.39	3.37	4.13	4.29

H17B	2.00	2.69	2.57	2.36	2.26
H19A	2.00	1.42	1.34	2.59	2.20
H19B	2.00	2.01	1.95	2.31	2.17
H19C	2.00	0.79	0.94	0.52	0.52

^{13}C	δ_{iso_exp} (ppm)	CB1	CB2	CB3	CB4
		δ_{iso_calc} (ppm)	δ_{iso_calc} (ppm)	δ_{iso_calc} (ppm)	δ_{iso_calc} (ppm)
C1	8.79	8.82	4.10	7.86	4.02
C2	131.88	145.93	132.33	145.32	131.69
C3	133.46	137.04	135.93	136.39	135.22
C4	127.85	120.25	135.00	119.69	134.81
C5	31.21	29.35	32.18	28.68	31.94
C6	36.02	37.49	35.27	36.42	34.89
C7	40.39	37.66	38.35	37.63	38.07
C8	160.61	159.59	157.71	159.39	156.59
C9	26.78	23.39	22.61	23.58	22.70
C10	119.68	119.32	122.80	119.07	122.41
C11	13.75	9.80	9.56	8.15	8.70
C12	131.88	138.19	138.57	122.77	122.52
C13	134.43	135.93	135.98	134.46	135.04
C14	124.50	126.72	127.12	143.50	143.75
C15	20.24	19.47	19.89	23.02	23.84
C16	27.66	26.91	27.10	27.16	28.06
C17	40.39	36.65	37.45	41.51	41.93
C18	160.61	157.16	158.22	158.61	157.73
C19	27.66	24.58	24.75	23.63	23.66
C20	119.00	122.60	121.92	120.00	119.13

^{15}N	CB1		CB2		CB3		CB4	
	δ_{iso_exp} (ppm)	δ_{iso_calc} (ppm)	δ_{iso_exp} (ppm)	δ_{iso_calc} (ppm)	δ_{iso_exp} (ppm)	δ_{iso_calc} (ppm)	δ_{iso_exp} (ppm)	δ_{iso_calc} (ppm)
N1	249.70	278.04	163.41	166.27	249.70	276.76	163.41	165.07
N2	163.41	152.19	249.70	254.94	163.41	153.03	249.70	255.06

N3	89.85	71.34	89.85	88.74	89.85	70.70	89.85	87.81
N4	78.35	85.31	78.35	79.21	78.35	91.73	78.35	84.39
N5	80.71	96.72	80.71	75.35	80.71	91.27	80.71	71.40
N6	178.87	177.95	178.87	173.60	178.87	170.78	178.87	168.13
N7	252.27	252.97	252.27	255.68	168.48	145.09	168.48	148.34
N8	168.48	168.27	168.48	171.82	252.27	282.61	252.27	287.64
N9	82.94	84.91	82.94	87.68	82.94	68.23	82.94	72.44
N10	77.37	81.03	77.37	78.32	77.37	82.45	77.37	76.45
N11	82.94	70.89	82.94	77.34	82.94	86.44	82.94	91.49
N12	184.20	169.49	184.20	180.11	184.20	170.05	184.20	180.88

Supplementary References

1 Middleton, D. A., Le Duff, C. S., Peng, X., Reid, D. G. & Saunders, D. Molecular Conformations of the Polymorphic Forms of Cimetidine from 13C Solid-State NMR Distance and Angle Measurements. *J. Am. Chem. Soc.* **122**, 1161-1170 (2000).

2 Oikawa, T., Okumura, M., Kimura, T. & Nishiyama, Y. Solid-state NMR meets electron diffraction: determination of crystalline polymorphs of small organic microcrystalline samples. *Acta Crystallogr. Sect. C* **73**, 219-228, (2017).

3 Lehmann, M. S., Koetzle, T. F. & Hamilton, W. C. PRECISION NEUTRON DIFFRACTION STRUCTURE DETERMINATION OF PROTEIN AND NUCLEIC ACID COMPONENTS. IV. The Crystal and Molecular Structure of the Amino Acid L-Histidine. *Int. J. Pept. Protein Res.* **4**, 229-239 (1972).
